# Supplementary material for: Distribution and Neurochemical Characterization of Dorsal Root Ganglia (DRG) Neurons Containing Phoenixin (PNX) and Supplying the Porcine Uterine Cervix
Source: Cells. 2025 Nov 23;14(23):1847. doi: 10.3390/cells14231847 (PMC12691474; doi:10.3390/cells14231847)
Supplement: Supplementary file 1 [file cells-14-01847-s001.zip › Supplementary - table S1.pdf]

Supplementary Table S1. List of primary antisera and secondary reagents used in the study: calcitonin gene related peptide (CGRP), calretinin (CRT), galanin (GAL), neuronal nitric oxide synthase (nNOS), pituitary adenylate cyclase-activating polypeptide (PACAP), phoenixin-14 (PNX), somatostatin (SOM), substance P (SP), fluorescein isothiocyanate (FITC), streptavidin (CY3).

Antigen	Code	Dilution	Host	Supplier
**Primary antibodies**				
CGRP	AB5920	1:10000	Rabbit	Merck Millipore, Temecula, CA, USA
CRT	6B3	1:2000	Mouse	SWANT, Switzerland
GAL	T-5036	1:2000	Guinea pig	Peninsula Laboratories, San Carlos, CA, USA
nNOS	N2280	1:7000	Rabbit	Merck Millipore, Temecula, CA, USA
PACAP	T-4465	1:25000	Rabbit	Peninsula Laboratories, San Carlos, CA, USA
PNX	H-079-01	1:6000	Rabbit	Phoenix Pharmaceuticals Inc, Burlingame, CA, USA,
SOM	MAB 354	1:50	Rat	Merck Millipore, Temecula, CA, USA
SP	8450-0004	1:200	Rat	Bio-Rad, Kidlington, UK
**Secondary reagents**				
CY3-conjugated anti-rabbit	711-166-152	1:1000	Donkey	Jackson I.R.; USA, Baltimore Pike
FITC-conjugated anti-mouse IgG	715-096-151	1:800	Donkey	Jackson I.R.; USA, Baltimore Pike
FITC-conjugated anti-guinea pig IgG	706-095-148	1:800	Donkey	Jackson I.R.; USA, Baltimore Pike
FITC-conjugated anti-rat IgG	712-095-153	1:400	Donkey	Jackson I.R.; USA, Baltimore Pike
