# Supplementary material for: Distribution and Neurochemical Characterization of Dorsal Root Ganglia (DRG) Neurons Containing Phoenixin (PNX) and Supplying the Porcine Uterine Cervix
Source: Cells. 2025 Nov 23;14(23):1847. doi: 10.3390/cells14231847 (PMC12691474; doi:10.3390/cells14231847)
Supplement: Supplementary file 1 [file cells-14-01847-s001.zip › Supplementary - table S2.pdf]

Supplementary Table S2. List of antigens used in pre-absorption test.

Antigen	Code	Dilution	Supplier
CGRP	T-4030	1:800	Peninsula Laboratories, San Carlos, CA, USA
CRT	6-His human calretinin (recombinant) Lot No.: 22	1:2000	SWANT, Switzerland
GAL	T-4862	1:1500	Peninsula Laboratories, San Carlos, CA, USA
nNOS	N3033	1:200	Sigma, St. Louis, MO, USA
PNX	079-01	1:7000	Phoenix Pharmaceuticals Inc; Burlingame; Kalifornia; USA
SOM	S9129	1:50	Sigma-Aldrich, St. Louis, MO, USA
SP	S6883	1:200	Sigma-Aldrich, St. Louis, MO, USA
